# Identifying and Characterizing the Poorest Urban Population Using National Household Surveys in 38 Cities in Sub-Saharan Africa

**DOI:** 10.1007/s11524-023-00805-z

**Published:** 2024-01-09

**Authors:** Fernando C. Wehrmeister, Leonardo Z. Ferreira, Agbessi Amouzou, Cauane Blumenberg, Cheikh Fayé, Luiza I. C. Ricardo, Abdoulaye Maiga, Luis Paulo Vidaletti, Dessalegn Y. Melesse, Janaína Calu Costa, Andrea K. Blanchard, Aluisio J. D. Barros, Ties Boerma

**Affiliations:** 1 Post-Graduate Program in Epidemiology, Universidade Federal de Pelotas, R Marechal Deodoro, 1160, 3 Piso.CEP 96020-220, Pelotas, RS Brazil; 2 International Center for Equity in Health, Universidade Federal de Pelotas, Pelotas, Brazil; 3 Institute for Global Public Health, University of Manitoba, Winnipeg, Canada; 4 Johns Hopkins University, Baltimore, MD USA; 5 Causale Consultoria, Pelotas, Brazil; 6 African Population and Health Research Centre, Dakar, Senegal; 7 Harvard University, Boston, MA USA

**Keywords:** Urbanization, Poverty, Sub-Saharan Africa

## Abstract

Identifying and classifying poor and rich groups in cities depends on several factors. Using data from available nationally representative surveys from 38 sub-Saharan African countries, we aimed to identify, through different poverty classifications, the best classification in urban and large city contexts. Additionally, we characterized the poor and rich groups in terms of living standards and schooling. We relied on absolute and relative measures in the identification process. For absolute ones, we selected people living below the poverty line, socioeconomic deprivation status and the UN-Habitat slum definition. We used different cut-off points for relative measures based on wealth distribution: 30%, 40%, 50%, and 60%. We analyzed all these measures according to the absence of electricity, improved drinking water and sanitation facilities, the proportion of children out-of-school, and any household member aged 10 or more with less than 6 years of education. We used the sample size, the gap between the poorest and richest groups, and the observed agreement between absolute and relative measures to identify the best measure. The best classification was based on 40% of the wealth since it has good discriminatory power between groups and median observed agreement higher than 60% in all selected cities. Using this measure, the median prevalence of absence of improved sanitation facilities was 82% among the poorer, and this indicator presented the highest inequalities. Educational indicators presented the lower prevalence and inequalities. Luanda, Ouagadougou, and N’Djaména were considered the worst performers, while Lagos, Douala, and Nairobi were the best performers. The higher the human development index, the lower the observed inequalities. When analyzing cities using nationally representative surveys, we recommend using the relative measure of 40% of wealth to characterize the poorest group. This classification presented large gaps in the selected outcomes and good agreement with absolute measures.

**Supplementary Information:**

The online version contains supplementary material available at 10.1007/s11524-023-00805-z.

## Introduction

In sub-Saharan Africa (SSA), an estimated 430 million people live in extreme poverty, encompassing one-third of the population [1]. Along with this very concerning scenario, urbanization in the SSA region is occurring much faster than elsewhere in the world [2]. This rapid increase is likely to have an impact on living conditions, especially among the poorest individuals [3, 4]. Knowing this group of people and their characteristics is essential for improving their living conditions and helping them to achieve their full potential for health and well-being.

Too little is known about the situation of the most socio-economically disadvantaged populations within the major cities in the SSA region [5, 6]. Household surveys are an imperfect yet under-utilized data source for cities and large metropolitan regions. They can provide information on health indicators, living standards, and educational indicators for many of the most populated cities in SSA. These surveys also allow the calculation of socioeconomic position measures using information on durable goods, household characteristics, or education. The most widely used is the wealth index, a relative measure of poverty based on assets and housing materials often divided into quintiles when wealth inequalities are under study [7]. However, other measures such as living below the poverty line [8], socioeconomic deprivation status [9], and the UN-Habitat slum classification [10, 11] can be also derived from surveys.

This paper has three main goals. The first is to identify and determine the most appropriate measure to distinguish poorer from richer populations in cities, the gap between the groups in selected outcomes, and the agreement between absolute and relative poverty measures. The second is to characterize the poorer and better-off populations in cities in terms of education and living standards, classifying them according to their performance, in terms of occurrence and inequalities (by poverty status), on selected outcomes. The third goal is to correlate outcomes estimates and inequalities with macro-level determinants, such as the human development index (HDI) and its domains.

## Methods

This study uses data from nationally representative surveys from sub-Saharan Africa. We used Demographic and Health Surveys (DHS) and Multiple Indicator Cluster Surveys (MICS) from 2010 onwards. Briefly, these are cross-sectional household surveys with similar sampling methods, using multi-stage clustering procedures to select women of reproductive age (15–49 years old) and their under-5 children. The surveys are nationally representative and allow reliable estimates of major cities or metropolitan regions.

We restricted the analyses to the largest city from each of 38 SSA countries’ urban areas. We considered the largest city the most populous city in the country if this city was a sampling domain in the survey. In some surveys, the largest city was not a sampling domain, and the metropolitan region or the region of the country which include the main city was chosen. These metropolitan regions were defined as having at least 70% of the population living in the major city [12]; we will refer to both as cities. More on DHS and MICS methods can be found elsewhere [7, 13].

### Definition of the Poor Population

#### Measures of Poverty

We explored several poverty measures to distinguish the urban poor from non-poor populations, all calculated at the household level. Although in most SSA countries the poverty levels are extremely high, for ease of reference, we refer to two groups as “poor” and “rich.” For relative measures, we focused on the wealth index, the standard measure in DHS and MICS surveys used to stratify analysis by socioeconomic position. It is calculated through a principal components analysis using a set of assets/goods (e.g., house, cars, motorcycle and computer ownership) and housing conditions (e.g., improved sources of water and sanitation, house materials) combined to generate a score for each household. We used the score from the original survey datasets because re-calculating the scores by restricting to the selected settings yielded correlation coefficients with the original measure of wealth above 0.95 in all surveys (data not shown). Then, we created four dichotomous poverty classifications using different cut-off points based on the percentile distribution of the wealth index: the first 30%, 40%, 50%, and 60% of the distribution were considered the poorest (while the remaining were considered richest). These measures are based on the relative position of a group within the wealth index, being known as relative measures of poverty.

For absolute measures of poverty, we used the following: people living below the poverty line (< US$ 1.9/day), the socioeconomic deprivation status (SDS), and the UN-Habitat slum classification. To estimate the population living below the poverty line, we used the predicted income proposed by Fink et al. [14]. This measure uses data from the gross domestic product (GDP), the Gini index and household expenditure to provide a predicted income for a given household, based on its position in the wealth index percentile. After that, we divided this predicted income by the number of household members and then divided it by 365.25 to represent the daily amount per household member. Additionally, we corrected it for household consumption expenditure, using publicly available data on the World Bank database (households and NPISHs final consumption expenditure (% of GDP) (https://data.worldbank.org/indicator/NE.CON.PRVT.ZS) and the final consumption expenditure (% of GDP) (https://data.worldbank.org/indicator/NE.CON.TOTL.ZS). This measure relies on several data sources, which can introduce some errors in its calculation. The number of household members can also affect, to a lesser extent, the precision of the income predictions. All the process was made for the entire country, as the proposed approach uses the country, and then restricted to the largest city in each country.

We also calculated the SDS [9], which is a multidimensional socioeconomic deprivation measure adapted from the Multidimensional Poverty Index [15, 16], allowing its calculation at the household level. It uses eight indicators, including two educational indicators (children in school age outside school and no household member aged 10 or more years with at least six years of education) and six living standards indicators (absence of electricity, improved sources of drinking water and sanitation facilities, poor household conditions, and the absence of a set of assets). The complete definitions for each included indicator are available elsewhere [9]. We used the UN-Habitat classification for a slum, which characterizes a household as within a slum if it lacks any of the following: durable housing material, sufficient living space, easy access to safe water, access to adequate sanitation, and security of tenure [11]. Information on the security of tenure is unavailable in DHS and MICS surveys; thus, it was ignored when creating the poverty classification, as recommended by the UN-Habitat.

It is important to highlight that these measures were selected as they can be extracted for surveys. We opted to keep those already known in the literature, and not to create new approaches to understanding poverty in the urban context.

#### Criteria to Define the Best Measure of Poverty

To identify the most adequate measure of poverty, we used three criteria to assess the feasibility of each poverty measure and its discriminatory power. Firstly, because sufficient sample sizes allow for more precise estimates, we examined each group’s unweighted sample size (absolute and relative numbers) for each poverty classification. The second criterion was to compare the median gap between poor and rich populations according to the outcomes described below. The last criterion evaluated the observed agreement between the relative (based on wealth index) and absolute measures of poverty, as some authors argued that relative measures are not comparable over different settings [14, 17].

### Selected Outcomes/Indicators

For our proposed analyses, we selected indicators based on two dimensions: (1) education in the household and (2) living standards. Two indicators derived from formal education were included based on the Multidimensional Poverty Index [16]. The first is defined as any school-aged child living in the household who is out of school. The second education indicator was based on any household member older than 10 years with less than 6 years of formal education. This choice was based on availability of the indicators on the surveys.

Three indicators were selected for the living standards dimension: absence of electricity, absence of improved drinking water sources, and absence of adequate sanitation facilities. The absence of electricity was defined as the household’s lack of access to electricity. Non-improved source of drinking water was defined as not having a safe drinking water source (e.g., unprotected well, unprotected spring, surface water) or safe but at a 30 min or longer walk distance in a roundtrip from home. Lack of adequate sanitation facilities refers to households using an unimproved sanitation facility (flush to elsewhere, pit latrine without a slab, bucket, hanging toilet, hanging latrine), no facilities, or improved but shared with others.

### City-Level Characteristics

We selected four macro-level city characteristics to examine the observed prevalence and inequalities in the selected outcomes among poor and rich populations. We used data from the Subnational Human Development Database [18] on the human development index (HDI), and its three separate dimensions (health, education, and income) all for cities or subnational regions. The HDI, which was developed by United Nations Development Programme (UNDP), aims to measure the well-being of a country considering those three dimensions [19]. The health index is the dimension related to life expectancy; the education index stands for the mean years of schooling for people aged 25 years or more; and the income index is based on the gross national income on a log scale. All these dimensions are standardized to create the HDI at subnational levels ranging from 0 to 1, with higher values representing better performance on each of the contextual factors selected. More details on these metrics can be found elsewhere [18]. Analysis of how the context affects estimates and poor-rich inequalities is essential to get a big picture of the inequalities and to consider solutions to narrow them.

### Statistical Analysis

For our first objective, to identify the most appropriate measure of poverty, we used equiplots to inspect the data and calculated the difference between the median values among all indicators in the poor and rich populations. We also calculated the observed agreement between the relative and absolute poverty measures, considering an agreement of 60% as adequate.

To describe the characteristics of poor and rich populations, the second objective of this study, we used the most adequate measure selected in the first part of this study. First, we described the prevalence among the poor population and the gap using boxplots to compare the distribution of the selected outcomes by the poverty groups. We also used equiplots to show the gap in the 38 selected cities across sub-Saharan Africa. The second set of analyses attempted to identify the best-performing cities, regarding the estimated occurrence and inequalities. For this, we defined cities as good, intermediate, and bad performers based on the prevalence and difference between groups. For this classification, we used the following cut-off points: low prevalence of the characteristic was defined as < 5%, implying good performance; intermediate prevalence was defined as 5–15%; and high prevalence was defined as > 15%. For inequalities, we used the difference between rich and poor groups and defined low inequalities as those differences between − 5 and 5 percentage points and high inequalities as those differences outside this range. Based on these two classifications, good performers were defined as low prevalence and low inequalities; intermediate performers were defined as intermediate prevalence and low inequalities or low prevalence and high inequalities; and cities with high or intermediate prevalence and high inequalities were defined as worse performers. This was defined because a low prevalence is expected for good performers when negative outcomes, such as the absence of electricity, are being analyzed.

Lastly, we calculated the Pearson correlation coefficient (ranging from − 1, perfect negative correlation, to + 1, perfect positive correlation) for the city-level characteristics (HDI, health, educational, and income indices) against the prevalence among poor and rich populations and the difference between rich and the poor estimates. For interpretation purposes, negative correlation coefficients mean lower inequality or prevalence with increasing of the HDI and its components.

All analyses were conducted using Stata® software version 17.0 (StataCorp LLC, College Stations, TX, USA) and accounted for each survey’s sample design.

## Results

### Identifying the Most Adequate Measure to Classify the Poorest and Richest Groups

We included 38 surveys (23 DHS and 15 MICS), which were carried out from 2010 (Burkina Faso) to 2021 (Niger). The median sample size among the poorest groups was lower when SDS (median 33, IQR 16 to 84) and people living below the poverty line (median 151, IQR 68 to 258) were analyzed. The highest median sample size among the poorest groups was observed for the UN-Habitat slum definition (median 771, IQR 544 to 976) and for the 60% of the wealth distribution (median 608, IQR 447 to 803) (Table 1). Supplementary Table 1 presents all data for each survey and city included.

**Table 1:** Summary statistics of the poorest group sample size, considering all 38 cities included

	Median		P25		P75		Minimum		Maximum	
Poverty measure	Abs. (*N*)	Rel. (%)	Abs. (*N*)	Rel. (%)	Abs. (*N*)	Rel. (%)	Abs. (*N)*	Rel. (%)	Abs. (*N*)	Rel. (%)
Poverty line (< USD 1.9 per day)	151	16.0	68	6.4	258	25.3	3	0.2	1,240	67.0
Socioeconomic deprivation status	33	3.5	16	1.6	84	7.7	1	0.1	214	19.1
UN-Habitat slum definition	771	71.3	544	64.2	976	78.0	185	42.8	1,674	93.8
Wealth score (30%)	337	33.1	244	31.7	455	34.2	110	27.2	719	35.9
Wealth score (40%)	403	39.6	294	38.4	544	40.5	134	33.6	872	42.5
Wealth score (50%)	509	49.4	373	48.3	679	50.8	167	43.8	1,061	52.5
Wealth score (60%)	608	59.3	447	58.1	803	60.7	200	53.4	1,267	62.8

**Fig. 1:**
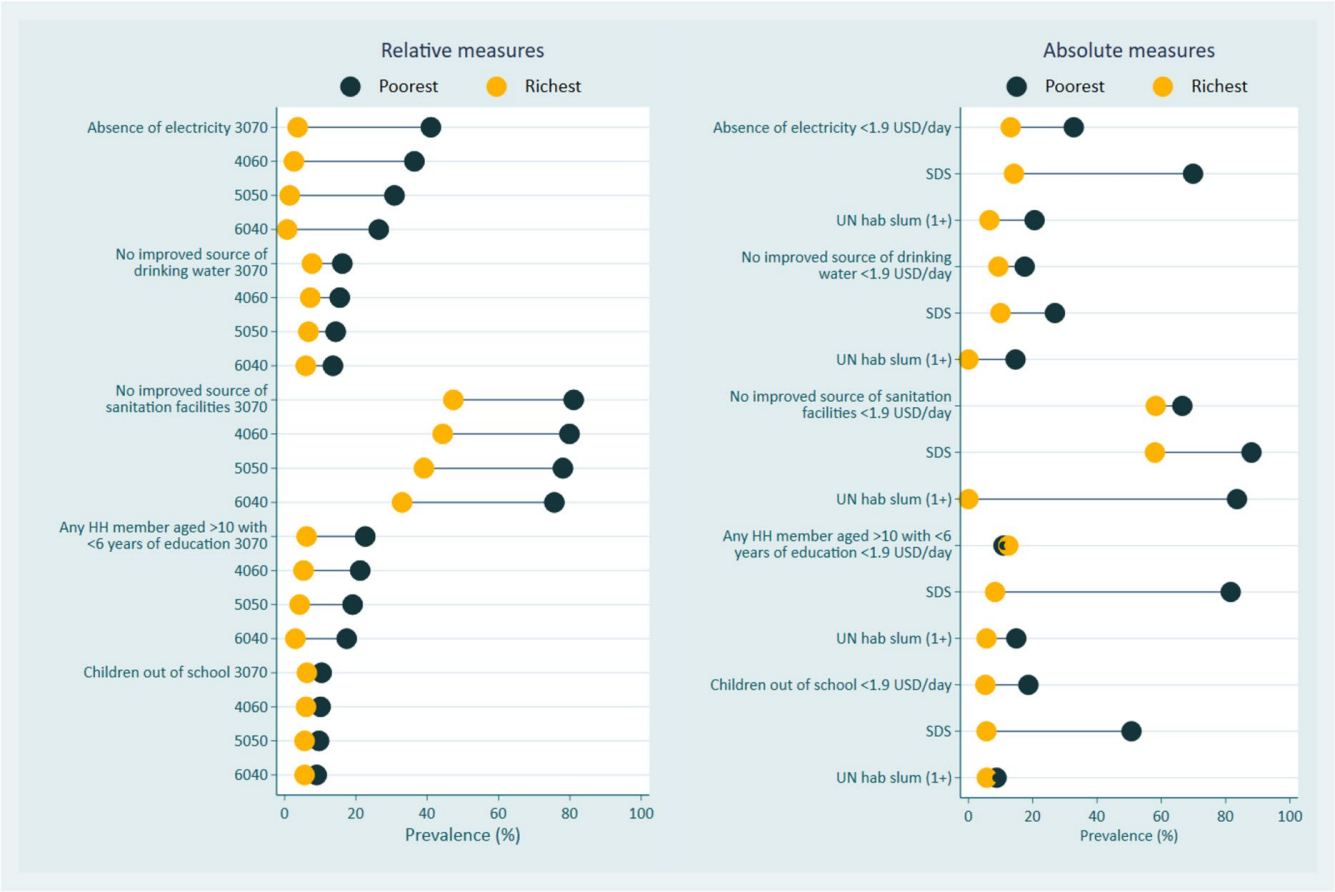
Equiplot showing the mean gap between poor and rich populations of all 38 cities included for all selected indicators

Figure 1 and Supplementary Table 2 show the mean prevalence and sample sizes among the poor and rich populations and the gap between them (considering all 38 cities) for all outcomes, according to relative and absolute poverty measures. Although SDS presented the higher gaps between poor and rich populations, comparing relative and absolute measures of poverty, the estimate for all indicators is based on a low sample size (average samples size = 52) while all relative measures rely on average sample sizes higher than 352 (for 30% poorest). Using the relative measures based on wealth distribution, the gap is consistent for all outcomes. The largest difference is observed with the cut-off point of 30% and 40%, except for the absence of improved sanitation facilities, where the gap was higher in the 60% cut-off point. The narrowest gap is observed for school-aged children out of school. Among the absolute measures, SDS showed the largest gaps for all indicators, except for the absence of sanitation facilities.

Supplementary Fig. 1 presents the distribution of observed agreement between the wealth index classifications and the absolute measures: poverty line, SDS, and UN-Habitat slum definition. The amplitude of agreement distribution is higher when relative measures are used compared to the UN-Habitat slum definition. Among the classifications based on the wealth index, the 40% poorest cut-off was the only measure that presented all median agreements with absolute measures above 60%. Therefore, we selected the classification based on the wealth index using a cut-off point of 40% for cross-cutting comparisons due to sufficiently large sample sizes, good discriminatory power between poor from rich populations for the selected outcomes, and highest agreement with absolute measures of poverty.

### Characterizing the Poor and Rich Populations using the Preferred Classification

In this part of the results and onwards, we will show only data considering the classification of the poorest based on 40% of the wealth distribution while the remaining were considered as richest. The prevalence distribution in each selected outcome according to poverty status is presented in Supplementary Fig. 2. The medians for all indicators are higher among the poor than the rich. The absence of electricity presented a much higher variability among the poor than the rich population. School-age children out of school was the least unequal indicator.

**Fig. 2:**
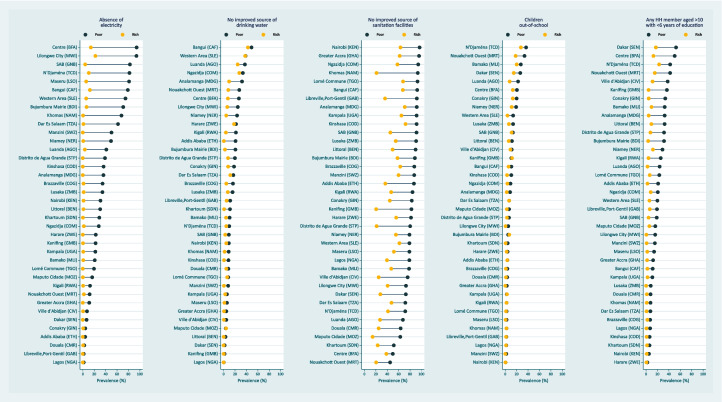
Prevalence of selected indicators by poor and rich populations in selected 38 major SSA cities, ordered by the prevalence in the poor population

Figure 2 shows the gap between poor and rich populations in each of the five selected indicators. Regarding the absence of electricity, Ouagadougou (Burkina Faso) (− 80.3 pp), Sector Autónomo de Bissau (Guinea-Bissau) (− 78.5 pp), Maseru (Lesotho) (− 75.2 pp), Lilongwe City (Malawi) (− 72.0 pp), and N’Djaména (Chad) (− 71.3 pp) were the most unequal of the 38 urban sites studied: the difference was larger than 70 percentage points in each city. Almost half of the poorest 40% of the population did not have improved sanitation facilities in all cities (median 82%, IQR 72 to 92%), and inequalities were larger in Windhoek (Namibia) (− 72.4 pp), Kanifing (Gambia) (− 61.8 pp), Distrito de Água Grande (São Tomé and Príncipe) (− 58.8 pp), and Libreville, Port-Gentil (Gabon) (− 55.8 pp). Regarding the educational indicators, household members with low schooling are the most unequal; Ouagadougou (− 37.2 pp), Dakar (Senegal) (− 35.2 pp), and Kanifing (− 32.2 pp) were the cities with large inequalities between rich and poor populations.

**Fig. 3:**
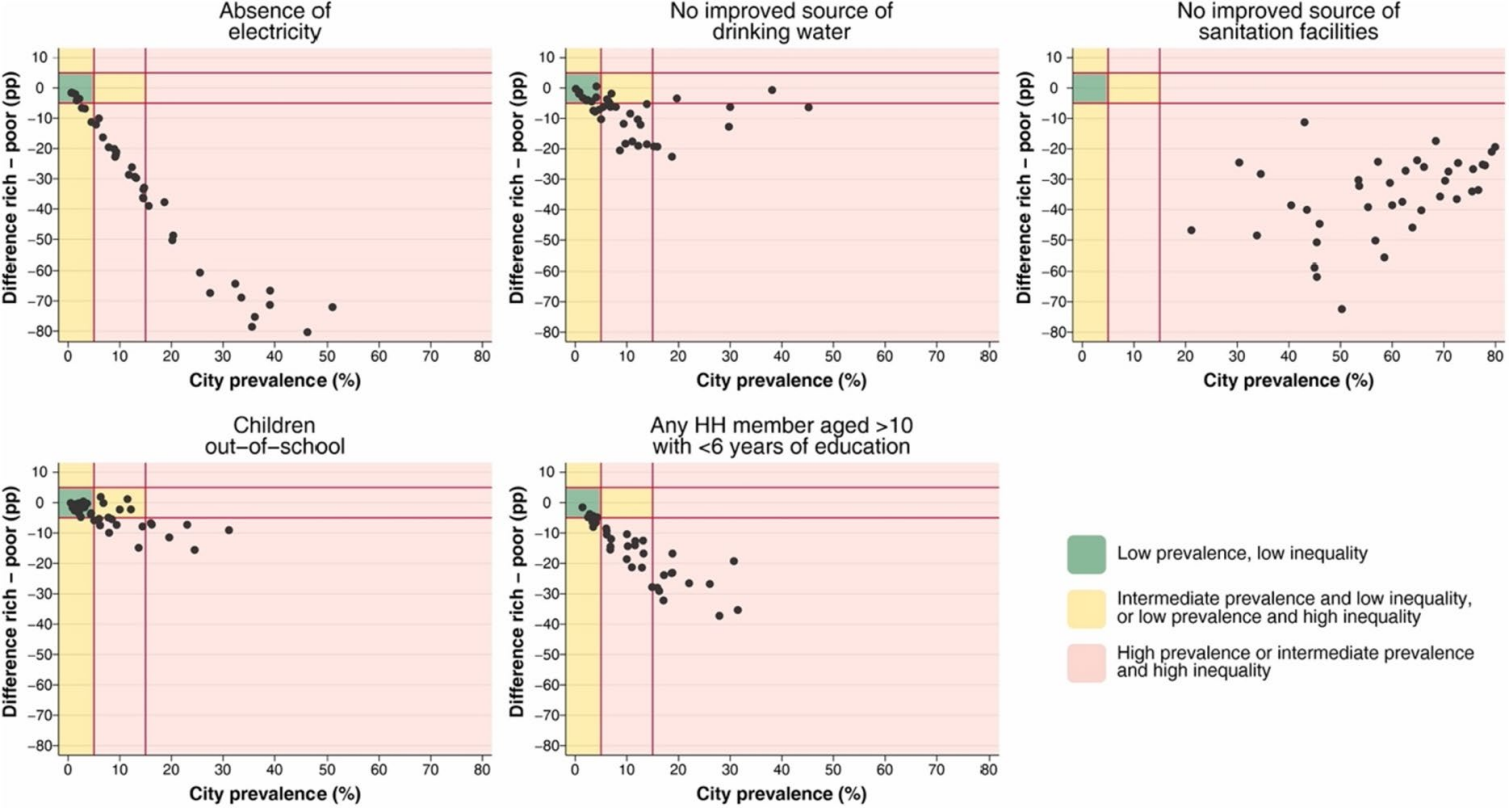
Quadrant plot showing good, intermediate, and worse performers in each of the five selected indicators**.** Note: Each dot represents a different city. HH, household

Figure 3 shows a quadrant plot with the difference between poor and rich populations and the prevalence of the outcome for the five selected indicators. For the absence of improved sanitation facilities indicator, all cities were classified as poor performers. Educational indicators showed more cities classified as good or intermediate performers compared to the indicators related to household conditions.

Table 2 shows each city according to its performance on the selected indicators. None of the cities was classified as a good performer on all five indicators. The worst performers were Luanda (Angola), Ouagadougou, N’Djaména, Ngazidja (Comoros), Analamanga (Madagascar), Bamako (Mali), Nouakchott (Mauritania), Niamey (Niger), and Freetown (Sierra Leone), while the best performers were Greater Accra (Ghana), Nairobi (Kenya), Douala (Cameroon), and Lagos (Nigeria).

**Table 2:**
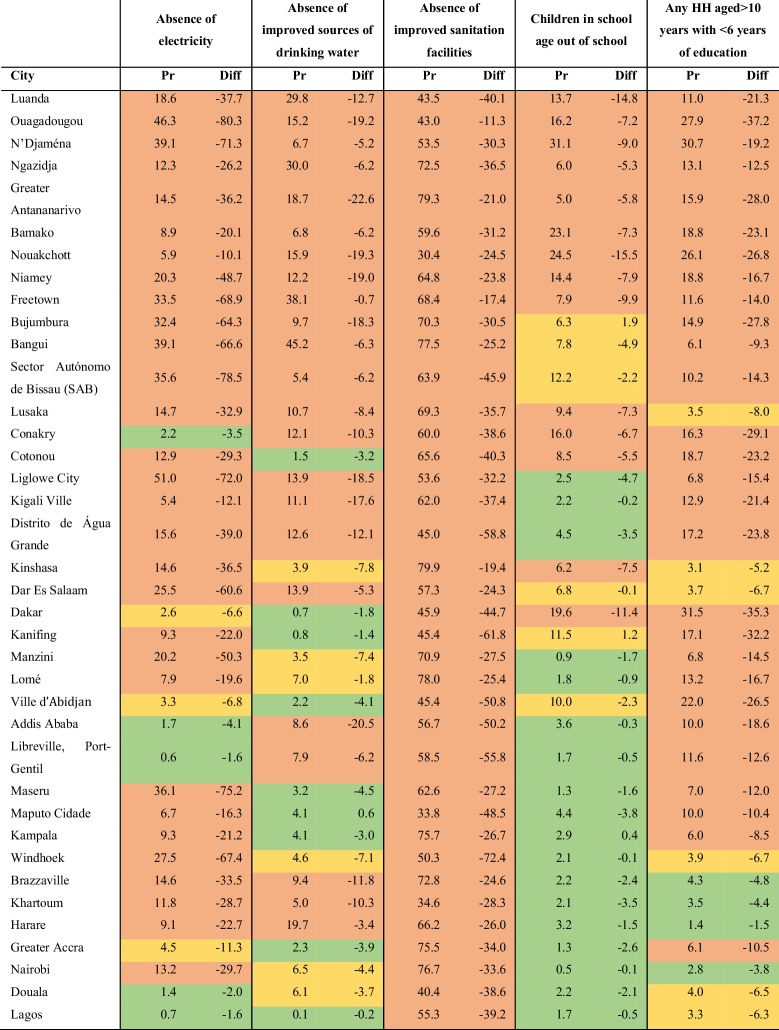
Classification of each city according to their performance on the selected outcomes

Note: Red-filled cells stand for worse performers; yellow-filled cells stand for intermediate performers, and green-filled cells stand for good performers. Also, “pr” stands for prevalence, and “diff” stands for difference calculated as rich-poor estimates

**Fig. 4:**
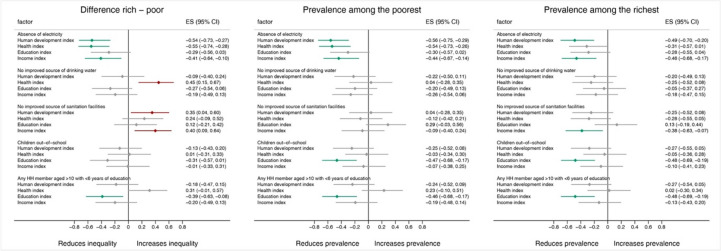
Correlation coefficients between the selected outcomes (coverage and inequalities among rich and poor populations) and city-level variables in selected cities. Note: HH, household

The correlation coefficients (and their 95% CI) between inequalities and prevalence among the poor and rich populations with city-level characteristics are shown in Fig. 4. In general, better performance in the city-level characteristics is correlated with lower inequalities and the prevalence of the selected outcomes. The health index is correlated with greater inequalities in the outcome of having no improved drinking water. The HDI and income index correlate with higher inequalities in the absence of improved sanitation facilities. The income index was correlated with a lower prevalence of improved sanitation only among the rich population.

## Discussion

Our results suggest that the most adequate classifications for poverty in major urban settings were those using a relative distribution of the wealth index. These relative measures presented the largest sample sizes, consistent gaps between the poor and rich groups, and a relatively high agreement with absolute poverty measures. Of all relative measures, using the cut-off at 40% for the poorest group was the best trade-off between sample size and the ability to differentiate the poorest from the richest populations in the cities in the context of a multi-country study using household survey data.

The choice of relative or absolute measures to classify socioeconomic groups has been the subject of much discussion. Some researchers have argued that absolute measures are preferable when multi-setting studies are being conducted [14, 17]. The strongest argument is that those classified as poor are based on the same metric in each setting. On the other hand, the criticism regarding relative measures emerges because the poorest in low-income settings could be completely different, in absolute terms, from the poorest in upper-middle-income settings. We used absolute and relative measures to define poverty and found a decent agreement between them. Insufficient sample sizes were found to be the major problem of absolute measures when assessing their feasibility for cross-cutting comparisons in large urban contexts. The SDS and people living below the poverty line classifications produce unrealistically small groups of poor households in most countries. On the other hand, the UN-Habitat definition of slums [11] tends to exaggerate and overclassify households as the poorest populations in the cities. In the context of the sub-Saharan Africa (SSA) region, it is expected that most of the households in large urban settings will have at least one slum characteristic. Finally, this measure targets slums rather than poor households, which despite the similarity are different definitions of poverty.

The world’s population has been shifting from rural areas to urban settlements, and over half now reside in urban settings [2]. While the process of urbanization is happening worldwide, the SSA region’s growth rate surpasses 4% a year, which has led to a doubling of its urban population at the beginning of the twenty-first century. At this pace, projections expect it to double again in the next 25 years [20]. This rapid population growth has not been accompanied by a similarly substantial increase in infrastructure in much of the SSA region, which can result in the poorer living in more adverse conditions than the richest in urban settings. The poorest in most cities were affected by the poor infrastructure in this analysis, as reflected in outcomes such as the absence of electricity and the absence of improved sanitation facilities—which presented the most prominent gaps and highest prevalence levels among the poorest populations. More marginalized neighborhoods, where the poorest groups tend to live [21], usually lack adequate infrastructure and unmet basic needs. The other outcomes analyzed here showed less marked inequalities, although important ones. On average, the out-of-school children outcome presented smaller but still noticeable gaps between poor and rich populations. School attendance also relies on infrastructure and may be affected by the number of schools close to the household and financial costs to buy school materials [22].

Using some city-level characteristics, we aimed to understand how macro-level aspects can be related to the gaps in and prevalence of urban characteristics within the city. In general, a better HDI is related to good performance in the prevalence and poor-rich inequalities on the selected outcomes. The HDI measures achievements in areas related to long life expectancy and living standards [19]. A study assessing water supply and HDI in Brazilian municipalities found that those municipalities with better water supplies have higher HDI means [23], similar to what we found in this study. Although it is not possible to determine the temporality between the measures assessed in our study, cities’ having higher HDI was correlated with fewer poor-rich inequalities. This could suggest that improving general living conditions will also reduce the gap between the socioeconomic groups in accessing these fundamental living standards.

This study has some limitations that should be highlighted. We aimed to show what could be done with publicly available household health surveys conducted in the context of major international survey programs, including DHS and MICS. We did not consider special surveys for cities or other sources of data such as censuses, routine health information systems, health and demographic surveillance studies [24–26], or specific research projects. These special surveys could provide a broader picture of the situation of the poor people living in urban areas compared to data from DHS and MICS. Also, using different measures, especially the absolute ones, could make outcome estimates unreliable since they could be based on very low sample sizes. We opted for crude dichotomous variables to classify the poor. This choice may obscure many of the adverse consequences and vulnerabilities associated with poverty and low socioeconomic position. However, we consider this as the best approach using national surveys. Large surveys, with large cities as sampling units, would be ideal for analyzing a wider spectrum of poverty because they would allow us to have more groups, showing an important socioeconomic gradient in the outcomes. We do not intend to discuss properties of poverty measures but to generate a big picture of the conditions of poor groups living in large cities context. Regarding the limitations of the indicators, both sets of indicators were selected due to the availability of surveys and could be part of the assessed measures of poverty. For example, the absence of improved sanitation facilities and drinking water sources are part of the wealth index. However, removing them from the original wealth score has little impact on inequalities [27]. The lengthy time frame over which surveys were conducted could reflect on levels of development, but huge changes are not expected on the indicators selected. The COVID-19 pandemic may also impact the results; the survey in Niger was carried out in 2021. The indicator that the pandemic could have more of an impact on is children out of school. As in this setting, all indicators show a consistent pattern; this impact seems minimal for overall results. Although these points, our results can broadly characterize the poor populations living in main cities.

In conclusion, our findings indicate that a poverty classification based on wealth, with a cut-off of 40%, has the most value when analyzing survey data in large urban settings. If the sample size allows, then a 30% cut-off can also be used. These measures are well correlated with absolute measures and can discriminate between the poor and rich populations in relation to a range of relevant socioeconomic outcomes. Cities with a higher HDI also tended to have lower inequalities in household conditions between the poor and rich within the city. When planning policies to reduce socioeconomic gaps, it will be essential to account for both individual and household, as well as macro-level characteristics, in cities where a growing proportion of people are living in sub-Saharan Africa.

## Supplementary Information

Below is the link to the electronic supplementary material.

Supplementary file1 (DOCX 339 KB)

## Data Availability

Data are publicly available in the DHS or MICS website.
