# Supplementary material for: Identifying and Characterizing the Poorest Urban Population Using National Household Surveys in 38 Cities in Sub-Saharan Africa
Source: J Urban Health. 2024 Jan 9;101(Suppl 1):5–17. doi: 10.1007/s11524-023-00805-z (PMC11602915; doi:10.1007/s11524-023-00805-z)
Supplement: Supplementary file 1 — Supplementary file1 (DOCX 339 KB) [file 11524_2023_805_MOESM1_ESM.docx]

Supplementary material

**Supplementary Table 1. Characteristics of the city settings with unweighted sample sizes for each poverty classification (percentage of total city sample in parenthesis)**

|  |  |  | **Poverty line (< 1.9 USD)** | **Socioeconomic deprivation status** | **UN-Habitat slum definition (1+ characteristic)** | **30% poor based on wealth** | **40% poor based on wealth** | **50% poor based on wealth** | **60% poor based on wealth** |
| --- | --- | --- | --- | --- | --- | --- | --- | --- | --- |
| **Country** | **Survey year** | **Urban setting** | **N (%)** | **N (%)** | **N (%)** | **N (%)** | **N (%)** | **N (%)** | **N (%)** |
| Angola | 2015 | Luanda | 212 (13.3) | 74 (4.7) | 1026 (64.2) | 531 (33.2) | 632 (39.5) | 787 (49.2) | 948 (59.3) |
| Benin | 2017 | Cotonou ^a^ | 82 (6.3) | 84 (6.7) | 932 (72.0) | 465 (35.9) | 550 (42.5) | 679 (52.5) | 803 (62.1) |
| Burkina Faso | 2010 | Ouagadougou ^a^ | 243 (25.9) | 117 (12.5) | 575 (61.2) | 297 (31.6) | 361 (38.4) | 456 (48.6) | 554 (59.0) |
| Burundi | 2016 | Bujumbura | 384 (40.9) | 90 (9.6) | 691 (73.5) | 276 (29.4) | 332 (35.3) | 429 (45.6) | 523 (55.6) |
| Central African Republic | 2018 | Bangui | 627 (43.4) | 133 (9.2) | 1275 (88.3) | 456 (31.6) | 551 (38.2) | 697 (48.3) | 843 (58.4) |
| Cameroon | 2018 | Douala | 77 (6.5) | 4 (0.3) | 644 (54.3) | 386 (32.5) | 467 (39.3) | 587 (49.5) | 704 (59.3) |
| Chad | 2019 | N'Djaména | 262 (23.4) | 214 (19.1) | 831 (74.3) | 324 (29.0) | 400 (35.7) | 510 (45.6) | 620 (55.4) |
| Comoros | 2012 | Ngazidja | 134 (13.8) | 41 (4.3) | 784 (80.9) | 269 (27.8) | 331 (34.2) | 424 (43.8) | 525 (54.2) |
| Congo Brazzaville | 2014 | Brazzaville | 1240 (67.0) | 21 (1.1) | 1420 (76.8) | 609 (32.9) | 738 (39.9) | 920 (49.7) | 1,111 (60.1) |
| Congo Democratic Republic | 2017 | Kinshasa | 464 (52.0) | 17 (1.9) | 837 (93.8) | 243 (27.2) | 300 (33.6) | 397 (44.5) | 476 (53.4) |
| Cote dIvoire | 2016 | Ville d'Abidjan | 161 (11.4) | 49 (3.5) | 819 (57.9) | 455 (32.2) | 544 (38.4) | 683 (48.3) | 833 (58.9) |
| Eswatini | 2014 | Manzini | 15 (3.3) | 6 (1.3) | 308 (68.1) | 158 (35.0) | 185 (40.9) | 211 (46.7) | 252 (55.8) |
| Ethiopia | 2019 | Addis Ababa | 22 (3.1) | 28 (4.0) | 492 (70.1) | 244 (34.8) | 293 (41.7) | 365 (52.0) | 435 (62.0) |
| Gabon | 2012 | Libreville, Port-Gentil | 399 (25.5) | 3 (0.2) | 1028 (65.8) | 525 (33.6) | 633 (40.5) | 797 (51.0) | 959 (61.4) |
| Gambia | 2019 | Kanifing | 241 (23.5) | 32 (3.2) | 533 (51.9) | 337 (32.8) | 405 (39.4) | 508 (49.5) | 609 (59.3) |
| Ghana | 2017 | Greater Accra | 65 (4.4) | 16 (1.1) | 1097 (74.8) | 502 (34.2) | 586 (39.9) | 721 (49.1) | 854 (58.2) |
| Guinea | 2018 | Conakry | 168 (17.0) | 48 (4.9) | 709 (71.8) | 342 (34.7) | 413 (41.8) | 512 (51.9) | 607 (61.5) |
| Guinea-Bissau | 2018 | Sector Autónomo de Bissau (SAB) ^b^ | 205 (22.6) | 94 (10.3) | 643 (70.7) | 302 (33.2) | 360 (39.6) | 448 (49.3) | 538 (59.2) |
| Kenya | 2014 | Nairobi | 20 (1.6) | 11 (0.9) | 986 (79.5) | 424 (34.2) | 508 (41.0) | 631 (50.9) | 753 (60.7) |
| Lesotho | 2014 | Maseru | 40 (6.4) | 9 (1.5) | 423 (67.5) | 199 (31.7) | 242 (38.6) | 305 (48.6) | 365 (58.2) |
| Madagascar | 2018 | Greater Antananarivo ^a^ | 140 (20.7) | 72 (10.7) | 584 (86.4) | 234 (34.6) | 274 (40.5) | 337 (49.9) | 401 (59.3) |
| Malawi | 2013 | Lilongwe City | 121 (25.3) | 37 (7.7) | 315 (65.9) | 170 (35.6) | 202 (42.3) | 250 (52.3) | 300 (62.8) |
| Mali | 2018 | Bamako | 232 (19.3) | 90 (7.6) | 839 (69.9) | 388 (32.3) | 468 (39.0) | 591 (49.3) | 716 (59.7) |
| Mauritania | 2019 | Nouakchott | 65 (3.7) | 141 (8.2) | 1132 (64.4) | 582 (33.1) | 690 (39.2) | 857 (48.7) | 1,025 (58.3) |
| Mozambique | 2015 | Maputo Cidade | 211 (36.1) | 16 (2.8) | 250 (42.8) | 177 (30.3) | 213 (36.5) | 267 (45.7) | 320 (54.8) |
| Namibia | 2013 | Windhoek ^a^ | 49 (5.5) | 22 (2.5) | 483 (54.4) | 300 (33.8) | 355 (40.0) | 441 (49.7) | 529 (59.6) |
| Niger | 2021 | Niamey | 369 (37.2) | 131 (13.4) | 785 (79.2) | 336 (33.9) | 400 (40.4) | 497 (50.2) | 587 (59.2) |
| Nigeria | 2016 | Lagos | 3 (0.2) | 1 (0.1) | 1,004 (63.8) | 532 (33.8) | 637 (40.5) | 782 (49.7) | 935 (59.4) |
| Rwanda | 2019 | Kigali Ville | 119 (10.0) | 39 (3.3) | 750 (63.2) | 356 (30.0) | 423 (35.6) | 536 (45.2) | 652 (54.9) |
| Sao Tome and Principe | 2019 | Distrito de Água Grande | 68 (9.3) | 25 (3.4) | 579 (78.9) | 249 (33.9) | 294 (40.1) | 373 (50.8) | 447 (60.9) |
| Senegal | 2019 | Dakar | 70 (21.3) | 30 (9.5) | 185 (56.4) | 110 (33.5) | 134 (40.9) | 167 (50.9) | 200 (61.0) |
| Sierra Leone | 2019 | Freetown ^a^ | 289 (13.9) | 154 (7.4) | 1,674 (80.6) | 719 (34.6) | 872 (42.0) | 1061 (51.1) | 1,267 (61.0) |
| Sudan | 2014 | Khartoum | 191 (25.3) | 12 (1.6) | 544 (72.0) | 240 (31.7) | 289 (38.2) | 362 (47.9) | 436 (57.7) |
| Tanzania | 2015 | Dar Es Salaam | 104 (14.9) | 12 (1.7) | 492 (70.3) | 228 (32.6) | 276 (39.4) | 348 (49.7) | 425 (60.7) |
| Togo | 2017 | Lomé Commune | 258 (23.5) | 38 (3.5) | 889 (81.0) | 360 (32.8) | 420 (38.3) | 521 (47.4) | 624 (56.8) |
| Uganda | 2016 | Kampala | 100 (8.0) | 22 (1.8) | 976 (78.0) | 407 (32.5) | 492 (39.3) | 606 (48.4) | 727 (58.1) |
| Zambia | 2018 | Lusaka | 414 (41.3) | 33 (3.3) | 757 (75.5) | 332 (33.1) | 401 (40.0) | 500 (49.9) | 595 (59.3) |
| Zimbabwe | 2019 | Harare | 29 (2.6) | 11 (1.0) | 825 (73.9) | 399 (35.8) | 472 (42.3) | 581 (52.1) | 688 (61.6) |

^a^ It is originally a region, which has over 70% of its population represented by the most populous city in the country.

^b^ Autonomous region that was considered as the most populous city in the country.

**Supplementary Table 2. Mean prevalence and difference between poor and rich subgroups in all 38 cities**

|  |  | **Prevalence** | | | **Sample size** | |
| --- | --- | --- | --- | --- | --- | --- |
| **Poverty classification** | **Indicator** | **Poor** | **Rich** | **Difference** | **Poor** | **Rich** |
| Wealth 60% | Absence of electricity | 27.1 | 0.7 | -26.4 | 636 | 443 |
|  | No improved source of drinking water | 13.9 | 6.1 | -7.8 | 636 | 443 |
|  | No improved source of sanitation facilities | 76.7 | 33.8 | -42.9 | 636 | 443 |
|  | Children out of school | 9.2 | 5.8 | -3.4 | 636 | 442 |
|  | Any HH member aged >10 with <6 years of education | 17.8 | 3.1 | -14.7 | 629 | 439 |
| Welath 50% | Absence of electricity | 31.6 | 1.5 | -30.1 | 530 | 549 |
|  | No improved source of drinking water | 14.7 | 6.9 | -7.8 | 530 | 549 |
|  | No improved source of sanitation facilities | 79.0 | 40.1 | -38.9 | 530 | 549 |
|  | Children out of school | 9.9 | 5.8 | -4.1 | 529 | 549 |
|  | Any HH member aged >10 with <6 years of education | 19.5 | 4.3 | -15.2 | 523 | 545 |
| Wealth 40% | Absence of electricity | 37.3 | 2.7 | -34.6 | 424 | 654 |
|  | No improved source of drinking water | 15.8 | 7.4 | -8.4 | 424 | 654 |
|  | No improved source of sanitation facilities | 80.7 | 45.5 | -35.2 | 424 | 654 |
|  | Children out of school | 10.3 | 6.2 | -4.1 | 424 | 654 |
|  | Any HH member aged >10 with <6 years of education | 21.6 | 5.4 | -16.2 | 419 | 649 |
| Wealth 30% | Absence of electricity | 42.0 | 3.8 | -38.2 | 354 | 725 |
|  | No improved source of drinking water | 16.6 | 7.9 | -8.7 | 354 | 725 |
|  | No improved source of sanitation facilities | 81.7 | 48.5 | -33.2 | 354 | 725 |
|  | Children out of school | 10.6 | 6.4 | -4.2 | 354 | 724 |
|  | Any HH member aged >10 with <6 years of education | 23.1 | 6.3 | -16.8 | 349 | 719 |
| UN-Habitat slum | Absence of electricity | 20.6 | 6.5 | -14.1 | 766 | 313 |
|  | No improved source of drinking water | 14.7 | 0 | -14.7 | 766 | 313 |
|  | No improved source of sanitation facilities | 83.5 | 0 | -83.5 | 766 | 313 |
|  | Children out of school | 8.8 | 5.7 | -3.1 | 765 | 313 |
|  | Any HH member aged >10 with <6 years of education | 14.9 | 5.6 | -9.3 | 757 | 310 |
| SDS | Absence of electricity | 69.9 | 14.2 | -55.7 | 52 | 1016 |
|  | No improved source of drinking water | 26.9 | 10.0 | -16.9 | 52 | 1016 |
|  | No improved source of sanitation facilities | 88.0 | 58.0 | -30.0 | 52 | 1016 |
|  | Children out of school | 50.7 | 5.6 | -45.1 | 52 | 1016 |
|  | Any HH member aged >10 with <6 years of education | 81.6 | 8.3 | -73.3 | 52 | 1016 |
| Poverty line (<1.9 USD/day) | Absence of electricity | 32.8 | 13.1 | -19.7 | 207 | 871 |
|  | No improved source of drinking water | 17.5 | 9.3 | -8.2 | 207 | 871 |
|  | No improved source of sanitation facilities | 66.5 | 58.3 | -8.2 | 207 | 871 |
|  | Children out of school | 18.7 | 5.3 | -13.4 | 207 | 871 |
|  | Any HH member aged >10 with <6 years of education | 10.9 | 12.4 | 1.5 | 207 | 861 |


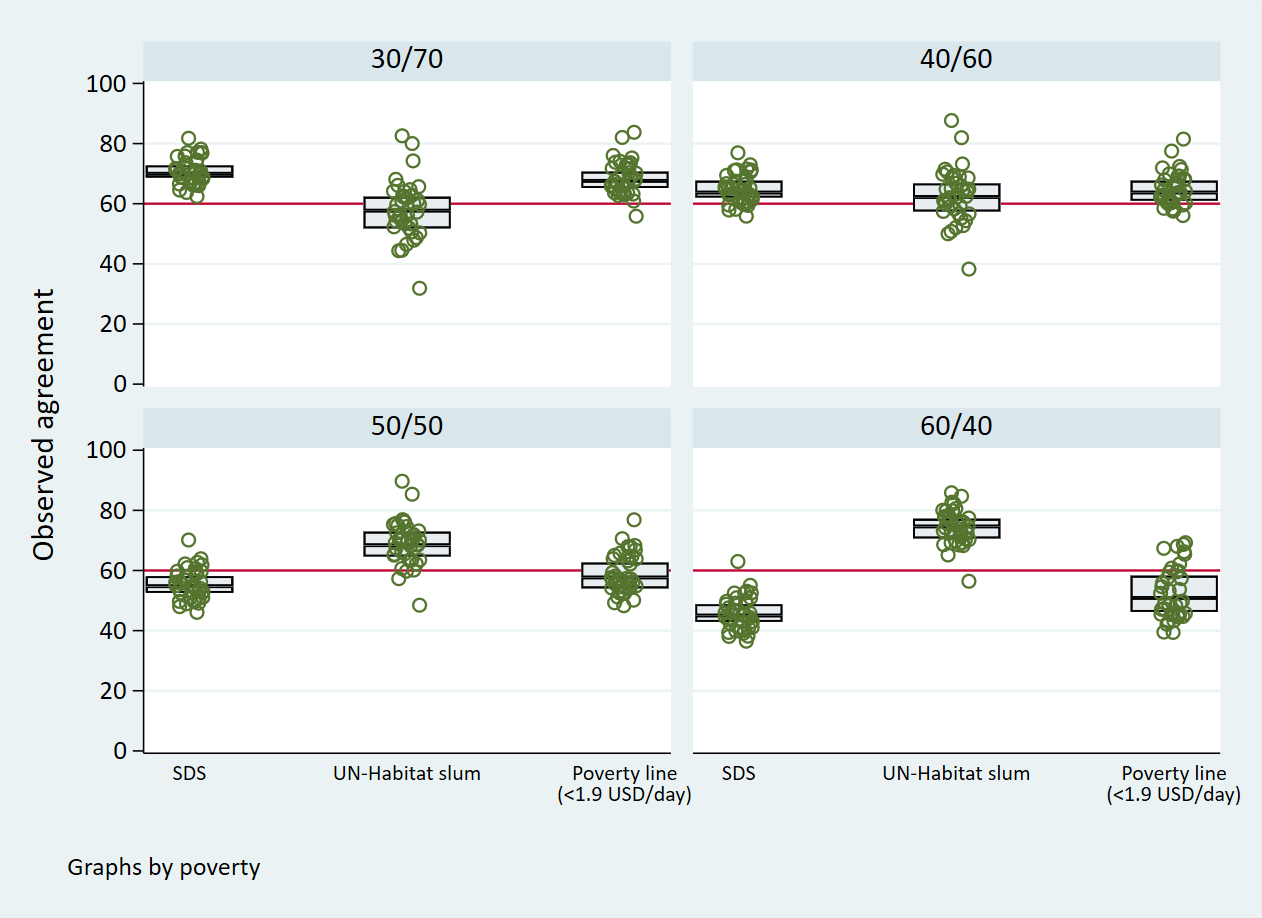


**Supplementary Figure 1. Median distribution of observed agreement between relative and absolute measures of poverty. Each dot represents one city.**


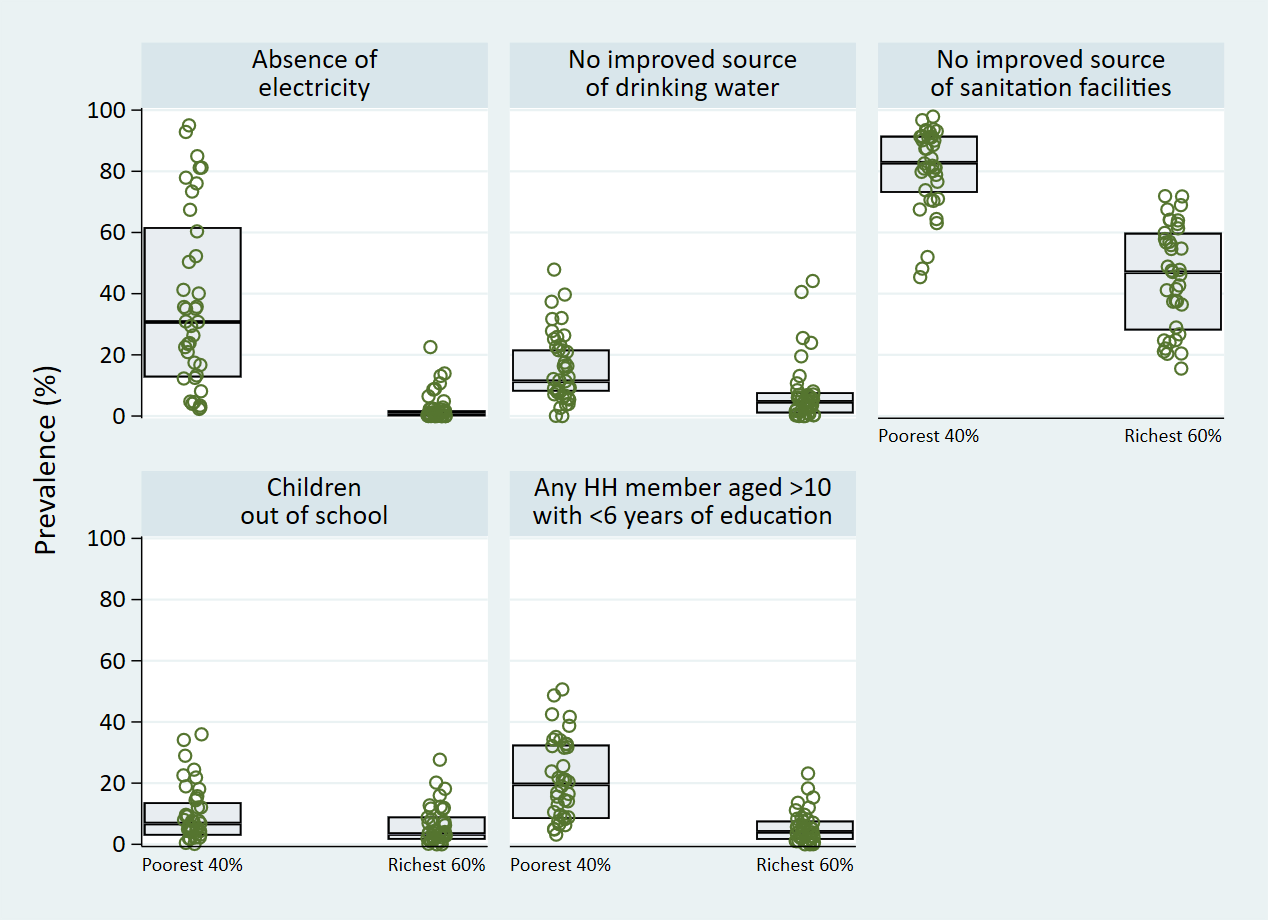


**Supplementary Figure 2. Median and distribution of countries (bar and dots, respectively) for selected outcomes among poor and non-poor groups. Each dot represents a city.**
